# An In Vitro Gut–Liver–Adipose Axis Model to Evaluate the Anti-Obesity Potential of a Novel Probiotic–Polycosanol Combination

**DOI:** 10.3390/foods14112003

**Published:** 2025-06-05

**Authors:** Simone Mulè, Rebecca Galla, Francesca Parini, Mattia Botta, Sara Ferrari, Francesca Uberti

**Affiliations:** 1 Department for Sustainable Development and Ecological Transition, University of Piemonte Orientale (UPO), 13100 Vercelli, Italy; simone.mule@uniupo.it (S.M.); rebecca.galla@uniupo.it (R.G.); 20032921@studenti.uniupo.it (F.P.); 20035008@studenti.uniupo.it (M.B.); sara.ferrari@uniupo.it (S.F.); 2 Noivita Srls, Spin Off, University of Piemonte Orientale (UPO), Via Solaroli 17, 28100 Novara, Italy

**Keywords:** lipid buildup, adipocyte metabolism, nutraceutical approach, novel combination, browning process, oral supplementation

## Abstract

The gut-liver-adipose axis plays a pivotal role in metabolic regulation, and its dysregulation contributes to obesity and metabolic syndrome. Probiotics and polycosanol have shown potential in modulating gut barrier integrity, lipid metabolism, and inflammation. This study aimed to evaluate their combined effects using an in vitro model of the gut-liver-adipose axis. Transwell^®^ system was used to recreate the interaction between intestinal (CaCo-2), hepatic (HepG2), and adipose (3T3-L1) cells. Cells were treated with *Bifidobacterium bifidum* GM-25, *Bifidobacterium infantis* GM-21, *Lacticaseibacillus rhamnosus* GM-28, and polycosanols. The effects were assessed by analyzing intestinal barrier integrity (TEER, tight junction proteins), hepatic and adipose lipid accumulation (Oil Red O staining), oxidative stress (ROS production, lipid peroxidation), inflammation (TNF-α) and lipid metabolism (CD36, PPARγ, AMPK and SREBP-1 levels). Probiotics and polycosanols improved intestinal integrity, increased butyrate production, and reduced ROS levels. Hepatic lipid accumulation was significantly decreased, with enhanced PPARγ and AMPK activation. In adipocytes, probiotic-polycosanols treatment suppressed SREBP-1 expression, enhanced lipid oxidation, and promoted UCP1 and PGC-1α expression, suggesting activation of thermogenic pathways. These findings underline a possible biological relevance of probiotics and polycosanols in modulating metabolic pathways, improving gut barrier integrity, and reducing inflammation, supporting their role as functional ingredients for metabolic health.

## 1. Introduction

Excessive fat buildup is a pathological condition known as obesity, which is usually measured by body mass index (BMI). BMIs over 25 suggest overweight, whereas those over 30 imply obesity. This illness is a significant risk factor for higher mortality from non-communicable diseases, such as type 2 diabetes, cardiovascular conditions, and several types of cancer. Obesity is a complex and complicated disorder impacted by both hereditary and acquired factors [1]. Cellular morphology changes characterise adipogenesis, triglyceride accumulation, and related gene expression [2]. Two important transcription factors involved in the early phases of adipocyte differentiation are peroxisome proliferator-activated receptor-γ (PPAR-γ) and CCAAT/enhancer-binding protein-α (C/EBP-α). Enzymes, including fatty acid synthase (FAS), adipocyte protein 2 (aP2), sterol regulatory element-binding protein-1c (SREBP-1c), and carnitine palmitoyltransferase-1 (CPT-1), control the formation of adipocytes [3]. New treatment approaches to decrease the socioeconomic effects of gastrointestinal-related liver illnesses need an understanding of gut-liver axis biological processes. The increased frequency of hepatic, gastrointestinal, and immune-mediated illnesses, including inflammatory bowel disease and autoimmune hepatitis, emphasises gut-liver interaction [4,5]. As a result, researchers are increasingly focusing on finding suitable tissues or in vitro models to explore the interplay between the gut and liver. Considering the liver receives its blood supply through the portal vein, the gut and visceral fat’s secretory profile directly influences hepatic metabolism (e.g., insulin sensitivity) [6]. Endotoxins from the stomach, pro-inflammatory cytokines, and free fatty acids (FFA) from visceral fat deposits in the portal vein may all be released more often in obese people. The existence of a real gut-liver-adipose axis can be determined according to these basic biological processes [7]. The liver and adipose tissue communicate through endocrine and metabolic signals. Adipose tissue releases adipocytokines, influencing insulin sensitivity and hepatic fat metabolism [8]. An excess of adipose tissue, typical of obesity, can lead to an increased release of free fatty acids into the blood, which accumulate in the liver, contributing to the development of hepatic steatosis [9]. At the same time, gut microbiota influences energy metabolism and body fat deposition [10]. Metabolites derived from gut bacteria, especially short-chain fatty acids (SCFA), regulate appetite control, lipid accumulation, and systemic inflammatory responses, influencing adipose tissue physiology [11]. The gut, liver, and adipose tissue interactions are significantly interdependent. A high-fat diet can alter gut microbiota composition, increase intestinal permeability, and induce hepatic inflammation. Lipid accumulation in hepatic and adipose tissues increases systemic inflammation, creating a loop that causes obesity and metabolic syndrome [12].

In this area, particular probiotics may adjust the intestinal microbiome alongside their effects on the immune system and metabolism [13]. For these reasons, probiotics may play an active role in modulating the immune response to pre-chronic low-level inflammation associated with metabolic syndrome [14]. The best-known and most-studied probiotics include *Lactobacillus acidophilus*, *Lactobacillus casei*, *Lactobacillus plantarum*, *Lactobacillus rhamnosus*, *Bifidobacterium bifidum*, and *Bifidobacterium longum*. Some probiotics can be used in functional meals or sold as supplements [15]. The gut microbiota is a significant factor that influences energy storage in the host and energy harvest from the diet. It significantly impacts host lipid and cholesterol metabolism [16]. In vitro research showed that *Bifidobacterium longum* subsp. *infantis* YB0411 (YB), which was selected using an in vitro adipogenesis test, has a function in the adipogenic differentiation of 3T3-L1 preadipocytes. YB administration significantly decreased tri-glyceride accumulation and expression of C/EBP-α and CCAAT/enhancer-binding protein β, and δ (C/EBPβ, and C/EBPδ), PPARγ, aP2, and acetyl-CoA carboxylase (ACC). YB administration also decreased the levels of basic autophagy markers such as Sequestosome 1 (p62) and microtubule-associated proteins 1A/1B light chain 3B (LC3β) in 3T3-L1 preadipocytes [17]. Similar findings were observed after treating 3T3-L1 preadipocytes with *Lacticaseibacillus rhamnosus* WB2804. These bacterial strains can tolerate simulated gastric conditions, adhere to intestinal cells, and prevent weight gain and metabolic abnormality from a high-fat, high-fructose diet [18].

In addition to the strong potential effects caused by probiotics, some natural extracts reveal anti-obesity effects, as reported in the literature [19,20,21]. Octacosanol, the primary constituent of Polycosanol, is a long-chain saturated fatty alcohol obtained from natural sources such as rice bran, wheat germ, sugar cane, and beeswax [22]. Dietary supplements, food additives, medicines, cosmetics, and additives for animal feed are just a few of the many uses for Polycosanol [23]. Previous studies have shown that it lowers blood cholesterol and reduces inflammation [24] and could represent a valid nutraceutical treatment, along with probiotics, for metabolic diseases without side effects. A Chinese group recently reported that the combination of polycosanol and atorvastatin in atherosclerosis patients could attenuate the statin-induced elevation of serum Proprotein Convertase Subtilisin/Kexin type 9 (PCSK9) levels [25]. This was accompanied by a reduction in serum total cholesterol and triglyceride contents, suggesting a potential effect of polycosanols on lipid composition [26]. Upon consuming polycosanol a further investigation demonstrated a substantial decrease in visceral fat content and systolic blood pressure [26]. It has been demonstrated that polycosanol is a natural product that is safe, well-tolerated, and effective against every aspect of metabolic syndrome [27]. Polycosanol is a potential therapeutic target for managing obesity and obesity-related metabolic diseases, according to a study that found it can boost adipose tissue’s energy expenditure [22,27,28,29]. For this reason, the project aimed to explore the biological interactions between the intestinal system, hepatic cells and adipose tissue, creating in vitro a gut-liver-adipose axis following the administration of a novel combination of probiotics and polycosanols. To ensure reproducibility and translational relevance, have been selected well-established cell lines: CaCo-2 (intestinal), HepG2 (hepatic), and 3T3-L1 (adipose). CaCo-2 cells provide a validated model of human intestinal absorption and barrier integrity, recognized by regulatory agencies for bioavailability predictions [30]. HepG2 cells, retain key hepatocyte-like functions, including lipid metabolism and drug detoxification, making them suitable for modelling steatosis and lipotoxicity [31,32]. Finally, 3T3-L1 preadipocytes, upon differentiation, express key adipogenic markers and reliably respond to metabolic stimuli, representing a robust system for studying lipid accumulation and browning effects [33,34]. This integrated and scalable co-culture system supports the mechanistic understanding of metabolic crosstalk underlying obesity, focusing on key markers of metabolism, lipid accumulation and lipid peroxidation such as cluster of differentiation 36 (CD36), PPARγ, glucagon-like peptide 1 (GLP-1) and Resistin (at liver compartment) and AMP-activated protein kinase (AMPK), sterol regulatory element-binding protein-1 (SREBP-1), Perilipin (at adipose level); with particular attention also to gut function and browning mechanism in 3T3-L1 adipocytes.

## 2. Materials and Methods

### 2.1. Agents Preparation

*B. bifidum* GM-25, *B. infantis* GM-21, and *L. rhamnosus* GM-28 were donated by Probionova SA (Lugano, Switzerland) and polycosanols (mainly composed of: 62% of Octacosanol; 17% of Triacontanol; 12% of Hexacosanol) were donated by Vivatis Pharma S.R.L. (Varese, Italy). All agents are prepared directly in the stimulation medium to reach the desired final concentration. Probiotics were dissolved in phenol red-free Dulbecco’s Modified Eagle’s Medium (DMEM, Merck Life Science, Rome, Italy) supplemented with 2 mM L-glutamine and devoid of fetal bovine serum (0% FBS, Merck Life Science, Rome, Italy) for each stimulation. A distinct product batch was utilised for each preparation to maintain consistency. Different concentrations of various freeze-dried probiotic powders, each possessing a specified colony-forming unit (CFU) content, were evaluated in a dose-response manner. Table 1 correlates in vitro dosages (mg) with their corresponding daily consumption in CFUs, ranging from 0.1 to 1.0 × 10^9^ CFU. Polycosanols were tested at concentrations ranging from 0.5 to 50 µg/mL [35]. CFU equivalents were computed using ISO 4833-1:2013 plate count procedures and manufacturer-validated CFU-to-milligram ratios [36,37]. Viable bacterial strain-specific cells were present per milligram of powder.

### 2.2. Cells Culture

CaCo-2 cells (American Type Culture Collection, ATCC, Manassas, VA, USA), a recognised in vitro model of intestinal epithelial cells [38,39,40,41,42], were grown in Dulbecco’s Modified Eagle’s Medium/Nutrient F-12 Ham (DMEM-F12, Merck Life Science, Rome, Italy) DMEM-F12 enriched with 10% fetal bovine serum (FBS), 2 mM L-glutamine, and 1% penicillin-streptomycin at 37 °C in a 5% CO_2_ atmosphere. To preserve the barrier’s integrity, cells ranging from passages 26 to 32 were utilised. Experiments involved seeding 2 × 10^4^ cells on 6.5 mm Transwell^®^ inserts (0.4 μm pores) for 21 days, with medium changes scheduled every other day. To replicate intestinal lumen and blood conditions, the apical and basolateral media pH were adjusted to 6.5 and 7.4 before stimulation [43,44].

HepG2 cells (ATCC, Manassas, VA, USA), originating from human hepatocellular carcinoma, were grown under standard conditions (37 °C, 5% CO_2_) [45] in DMEM supplemented with 10% FBS, 2 mM L-glutamine, and 1% penicillin-streptomycin (Merck Life Science). Repeatability was ensured by selecting 90–95% confluent cultures [44]. In dose-response investigations, 3.5 × 10^4^ cells were cultured in 24-well plates with 6.5 mm Transwell^®^ inserts and monolayer maturity was validated by trans-epithelial electrical resistance (TEER) measuring 486 Ω·cm^2^ [46]. Additionally, 1 × 10^5^ cells were grown in 6-well plates and subjected to CaCo-2 supernatants for 24 h before Enzyme-Linked Immunosorbent Assay (ELISA) analysis. Retaining their metabolic and inflammatory functions, cells were utilized in an undifferentiated state [47]. The pathological transition (steatosis condition) was simulated by treatment for 48 h with oleic acid 50 μM [48].

3T3-L1 preadipocytes (ATCC, Manassas, VA, USA) were cultured in DMEM supplemented with 10% FBS, 2 mM L-glutamine, and 1% penicillin-streptomycin at 37 °C and 5% CO_2_. To maintain adipogenic potential [49], cells were subcultured at passages 2–10 before reaching 70% confluence [50]. For differentiation, 1 × 10^5^ cells per well were inoculated in 6-well plates and permitted to proliferate to confluence over 2 days. DMEM with 10% FBS, 0.5 mM 3-isobutyl-1-methylxanthine, 1 μM dexamethasone, and 10 μg/mL insulin was used to promote differentiation on day 0 for 48 h. From days 2 to 4, cells were grown in DMEM with 10% FBS and 10 μg/mL insulin. New media was added on day 4, and DMEM with 10% FBS was added on day 6. Differentiation ended on day 8 [48]. Differentiated adipocytes were used in dose-response tests to study adipogenesis, lipid metabolism, accumulation, and indicators of brown adipose tissue (BAT)-like properties [51,52].

### 2.3. Experimental Protocol

**Figure 1:**
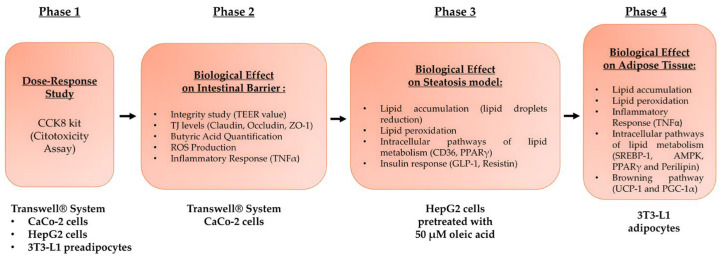
Flow diagram of the various phases constituting the experimental protocol and the analyses carried out on the intestine-liver-adipose tissue axis.

The research comprised four stages (Figure 1). In the first phase, a Transwell^®^ device was used to simulate the gut-liver-adipose tissue axis and determine appropriate doses of three probiotics and polycosanols. The apical Transwell^®^ membrane was seeded with CaCo-2 cells to generate a mature monolayer. The basolateral medium containing intestinal metabolites was administered to HepG2 cells for 24 h after 6 h of stimulation with each test agent. The hepatic metabolites were subsequently collected and utilised to treat 3T3-L1 preadipocytes that had been cultured in 6-well plates for 24 h. The most effective agent concentrations for subsequent experiments were determined by evaluating cell viability using the Cell Counting Kit-8 (CCK8).

In the second phase, a three-dimensional intestinal barrier model was established by inoculating CaCo-2 cells onto the apical surface of Transwell inserts, and the cells were then subjected to stimulation with the test chemicals. To prevent irritation from probiotics and polycosanol, the integrity of the barrier and the levels of tight junction (TJ) proteins were measured. Metabolite cross-bordering and probiotic adherence to the intestinal epithelium were also assessed [53].

In phase three, HepG2 cells were primed with 50 µM oleic acid for 48 h to induce metabolic dysfunction [48], followed by a 24-h treatment with intestinally metabolised probiotic substances, with or without polycosanols. These effects were assessed in terms of hepatic lipid accumulation, intracellular triglycerides, lipid metabolism indicators (CD36, PPARγ), and insulin signalling mediators (GLP-1, Resistin).

The concluding phase focused on adipose tissue using 3T3-L1 preadipocytes, which underwent complete differentiation into adipocytes over a period of eight days. Throughout the differentiation phase, these cells were exposed to hepatic metabolites resulting from probiotic therapy for 24 h, both in the presence and absence of polycosanols. The purpose was to assess the possible lipid-lowering effects and the suppression of adipogenesis. Lipid accumulation, intracellular triglyceride levels, and the activation of intracellular pathways related to lipid metabolism, such as SREBP-1, Perilipin, PPARγ, and AMPK, were evaluated to achieve this. The inflammatory response was assessed via TNFα analysis and lipid peroxidation measures. Additionally, uncoupling protein 1 (UCP1) and Peroxisome Proliferator Activated Receptor 1 Alpha (PGC-1α), two key markers associated with the transformation of white adipose tissue (WAT) into a brown-like phenotype, were investigated to describe the ability of probiotics and polycosanols to decrease fat accumulation and promote thermogenesis by enhancing fat oxidation at the cellular level.

### 2.4. In Vitro Intestinal Barrier Model

A gut epithelial barrier model was established utilising CaCo-2 cells to assess the integrity of the intestinal monolayer before drug translocation. TEER was assessed bi-daily over 21 days using an Epithelial Volt/Ohm meter (EVOM3) equipped with STX2 electrodes (World Precision Instruments, Sarasota, FL, USA). Full monolayer maturation was indicated by values of ≥400 Ω·cm^2^ [54,55,56]. To replicate luminal and circulatory conditions, pH levels were adjusted to 6.5 (apical) and 7.4 (basolateral) on day 21 [53]. The apical pH was adjusted using 0.1 N HCl or NaOH, buffered with 10 mM HEPES and verified with a calibrated digital pH meter (pH50 VioLab pH meter with XS 201T DHS digital electrode, XS Instruments, Modena, Italy). Following 6 h of treatment, the pH remained stable within ±0.2 units. TEER was re-measured after 15 min at 37 °C and 5% CO_2_. Following collection, the basolateral media was added to HepG2 cells for further examination.

### 2.5. Cell Counting Kit-8 (Cytotoxicity Assay)

Cell viability was evaluated using the Cell Counting Kit-8 (CCK-8; Merck Life Science, Rome, Italy) [57]. CaCo-2 (2 × 10^4^) and HepG2 (3.5 × 10^4^) cells were seeded in 6.5 mm Transwell^®^ inserts (Corning^®^ Costar^®^, Merck Life Science, Rome, Italy) and pre-incubated for 24 h at 37 °C, 5% CO_2_. Simultaneously, 3T3-L1 preadipocytes (1 × 10^5^) were plated in 6-well plates under the same conditions. After incubation, CaCo-2 cells were treated for 6 h, and HepG2 and 3T3-L1 cells for 24 h with all the agents. Subsequently, 10 µL of CCK-8 reagent was added to each well and incubated for 1–4 h. Absorbance was recorded at 450 nm using a microplate reader (Infinite 200 Pro MPlex, Tecan, Männedorf, Switzerland).

### 2.6. Analysis of ZO-1

ZO-1 concentrations in CaCo-2 lysates were quantified with a MyBioSource (San Diego, CA, USA) ELISA kit according to the manufacturer’s guidelines [58]. Following two freeze-thaw cycles and a wash with cold phosphate buffer saline (PBS), lysates were centrifuged at 5000× *g* for 5 min at 4 °C. Samples (100 μL) were incubated on ELISA plates at 37 °C for 90 min. Washing was followed by 45-min incubations at 37 °C of Detection Solutions A and B. After that, the substrate was added, and it was incubated for 20 min at 37 °C in the dark. The reaction was terminated with 50 μL of the stop solution, and the absorbance was recorded at 450 nm utilising a Tecan Infinite 200 Pro MPlex. According to a 0–1000 pg/mL standard curve, ZO-1 levels were normalised to total protein by BCA assay (Thermo Fisher Scientific, Waltham, MA, USA). Results are shown as pg/mL per μg protein and percentage increase compared to controls, from five separate triplicate experiments.

### 2.7. Analysis of Claudina 4

Claudin-4 levels in CaCo-2 lysates were measured using a Cusabio ELISA kit (Huston, TX, USA) according to the manufacturer’s protocol [58]. Lysates were centrifuged, and 100 μL samples were incubated in ELISA plates at 37 °C. After washing, biotinylated antibody and HRP-avidin were added sequentially, followed by incubation steps. TMB substrate was applied, incubated in the dark, and the reaction was stopped. Absorbance at 450 nm was recorded, and concentrations were calculated using a standard curve ranging from 0 to 1000 pg/mL, normalised to total protein by BCA assay (Thermo Fisher Scientific, Waltham, MA, USA). Results are expressed as pg/mL per μg protein and as a percentage increase versus control from five replicate experiments.

### 2.8. Analysis of Occludin

Occludin concentrations in CaCo-2 lysates were quantified with a human occludin ELISA kit (MyBioSource, San Diego, CA, USA) in accordance with the manufacturer’s instructions [58]. Using 1× PBS, cells were lysed, centrifuged at 1500× *g* for 10 min at 4 °C, and 100 μL of each lysate was incubated in assay strips at 37 °C for 90 After removing the supernatant, strips were repeatedly incubated with 100 μL of detection solution A and B for 45 min at 37 °C, with washing steps in between. Next, add 90 μL substrate solution and incubate at 37 °C for 20 min in the dark. Absorbance was measured at 450 nm using an Infinite 200 Pro-MPlex spectrophotometer (Tecan, Männedorf, Switzerland) after stopping the reaction with 50 μL stop solution. Odcludin concentrations were adjusted to total protein assessed by BCA assay (Thermo Fisher Scientific, Waltham, MA, USA) and compared to a standard curve. Data from five independent triplicate tests are shown as pg/mL per μg protein and percentage increase compared to controls.

### 2.9. Surface Hydrophobicity Assay

The surface hydrophobicity experiment was conducted following established protocols [59]. To facilitate phase separation, 4 mL of the probiotic suspension and 0.4 mL of xylene were combined for the test and incubated for 15 min. The negative control was PBS. Both the aqueous phase and control had 600 nm absorbance. Ten samples were examined in parallel for each condition in triplicate. The following formula calculates the hydrophobicity of the surface:

(1) $$\mathrm{Surface}\;\mathrm{hydrophobicity}\;=\;\frac{{OD}_{600}\left(control\right)-{OD}_{600}\left(test\right)}{{OD}_{600}(control)}\times 100\%$$

OD_600_ (control): the absorption value of the control.

OD_600_ (test): the absorption value of the sample.

### 2.10. Reactive Oxygen Species (ROS) Production

ROS generation was evaluated by measuring the release of superoxide anion during CaCo-2 cell stimulation [60]. Both treated and untreated samples were incubated with 100 µL of cytochrome C and 100 µL of superoxide dismutase for 30 min. All reagents were sourced from Merck Life Science, Rome, Italy. Absorbance at 550 nm was measured utilizing an Infinite 200 Pro MPlex spectrophotometer (Tecan, Männedorf, Switzerland). Superoxide production was measured as nanomoles of reduced cytochrome C per microgram of protein, normalized to control levels, and expressed as a percentage increase from five independent triplicate assays.

### 2.11. TNFα Quantification Assay

TNFα levels in CaCo-2 and 3T3-L1 adipocyte supernatants were measured using a TNFα ELISA kit (Merck Life Science, Rome, Italy) according to the manufacturer’s protocol [61]. Supernatants collected after cell stimulation were analysed by measuring absorbance at 450 nm using an Infinite 200 Pro MPlex (Tecan, Männedorf, Switzerland). Concentrations were determined using a standard curve (24.58–6000 pg/mL) and expressed as a percentage increase relative to the control. Data represent the mean of five independent experiments performed in triplicate.

### 2.12. Butyric Acid Quantification Assay

Following the manufacturer’s procedure, an ELISA kit (Cloud-Clone, Wuhan, China) was used to assess butyric acid concentrations in the basolateral compartment of the Transwell^®^ system [62]. Absorbance was measured at 450 nm using an Infinite 200 Pro MPlex plate reader (Tecan, Männedorf, Switzerland) after the addition of the stop solution. The detection range was from 123.5 to 10,000 pg/mL, and concentrations were estimated by interpolation against a standard curve ranging from 0 to 10,000. The assay detection limit was below 55.5 pg/mL. Results are presented as mean pg/mL values obtained from five independent tests carried out in triplicate.

### 2.13. Lipid Accumulation Assay

Using the Adipogenesis Assay Kit (Cayman Chemical, Ann Arbour, MI, USA), lipid accumulation in HepG2 cells and 3T3-L1 adipocytes was measured according to the manufacturer’s instructions. After treatment, the cells were fixed for 15 min with 75 μL of fixative and washed twice with 100 μL of wash buffer. An Oil Red O solution (75 μL) was used to stain lipids for 20 min at room temperature, and they were visualised with a Leica DM1000 microscope (Leica Microsystems Srl, Milan, Italy) at 10× (100 μm) objective and representative images were obtained for the analysis of the density of lipid droplets using ImageJ software, version 1.54p (Appendix A). Each well was treated with 100 μL of dye extraction solution and gently stirred for 30 min for quantification. Absorbance was quantified at 490–520 nm utilising an Infinite 200 Pro MPlex microplate reader (Tecan, Männedorf, Switzerland). Based on five separate triplicate tests, results were expressed as percentage changes relative to untreated control cells (0%).

### 2.14. Thiobarbituric Acid Reactive Substances Test (TBARS)

Lipid peroxidation in HepG2 and 3T3-L1 adipocyte lysates was evaluated using the TBARS assay (Cayman Chemical, Ann Arbour, MI, USA) [63]. Following treatment, cells were fixed with 75 μL of fixative for 15 min, washed twice with 100 μL of wash solution, and stained with 75 μL of Oil Red O working solution for 20 min at room temperature. Lipid droplets were then examined using a Leica DM1000 microscope. For quantification, 100 μL of dye extraction solution was added to each well, and gently mixed for 30 min, and the absorbance was measured at 490–520 nm using an Infinite 200 Pro MPlex plate reader (Tecan, Männedorf, Switzerland).

### 2.15. CD36 ELISA Kit

CD36 concentrations in HepG2 cell culture supernatants were quantified utilising the Human CD36/SR-B3 ELISA Kit (Thermo Fisher, Milan, Italy). Following stimulation, 100 μL of the collected supernatants were dispensed into the wells of an ELISA plate and incubated on a shaker at room temperature for 150 min. After that, 100 μL of biotin-conjugate was added and incubated for 60 min. After four washes, 100 μL streptavidin-HRP was administered for 45 min. The wells were subsequently incubated with 100 μL of TMB substrate in the dark for 30 min. Absorbance was measured at 450 nm using a Tecan Infinite 200 Pro MPlex microplate reader (Männedorf, Switzerland) after the reaction was stopped with 50 μL of the stop solution. CD36 concentrations were measured from a standard curve ranging from 2 to 500 ng/mL. Results are percentage changes relative to controls from five independent triplicate experiments.

### 2.16. PPARγ ELISA Kit

The transcriptional activity of PPARγ in nuclear extracts from HepG2 cells was assessed using the PPAR Gamma Transcription Factor Assay Kit (Abcam, Cambridge, UK) according to the manufacturer’s instructions. The nuclear fraction was isolated by centrifuging cells in a nuclear extraction solution with protease and phosphatase inhibitors. The extracts were treated with primary antibodies against PPARα and PPARγ, followed by incubation with secondary antibodies. Absorbance was quantified at 450 nm using a Tecan Infinite 200 Pro MPlex reader (Männedorf, Switzerland). Data represent mean values from five separate triplicate assays, expressed as a percentage increase above untreated controls.

### 2.17. Resistin ELISA Kit

Resistin concentrations in the supernatants of HepG2 cells were quantified utilising the Resistin Human ELISA Kit (Thermo Fisher, Milan, Italy). After cell stimulation, the supernatants were incubated with 50 μL of sample and 50 μL of biotin-conjugate per well for 120 min on a microplate shaker at room temperature. After four washes, 100 μL of Streptavidin-HRP solution was added and incubated at room temperature for 60 min. After four washes, 100 μL of TMB substrate was added, and the reaction was incubated in the dark for 10 min before being stopped with 100 μL of stop solution. Absorbance was measured at 450 nm utilising an Infinite 200 Pro MPlex spectrophotometer (Tecan, Männedorf, Switzerland). Resistin concentrations were estimated from a standard curve ranging from 31 to 2000 pg/mL. Data is expressed as percentage increases compared to controls, obtained from five separate experiments performed in triplicate.

### 2.18. GLP-1 ELISA Kit

GLP-1 activity in HepG2 cell culture supernatants was measured using the Human GLP-1 (1-37a) ELISA Kit (Thermo Fisher, Milan, Italy) following the manufacturer’s protocol. Briefly, 100 μL of each sample was added to ELISA plate wells and incubated for 150 min at room temperature with shaking. Subsequently, 100 μL of biotin-conjugate and then 100 μL of Streptavidin-HRP were added, followed by 100 μL of TMB substrate in the dark. After stopping the reaction with 50 μL of stop solution, the absorbance was measured at 450 nm using a Tecan Infinite 200 Pro MPlex spectrophotometer (Männedorf, Switzerland). Concentrations of GLP-1 were determined using a standard curve from 2.3 to 150 pg/mL. The results indicate the percentage increase compared to control samples and are derived from five independent experiments conducted in triplicate.

### 2.19. SREBP-1 Detection Assay

Following stimulation, 3T3-L1 adipocytes were lysed to measure SREBP-1 levels according to the ELISA kit protocol (LSBio, Seattle, DC, USA). Aliquots of 100 μL were loaded into 96-well ELISA plates and incubated at 37 °C for 2 h. Next, 100 μL of Detection Reagent A was added, followed by a 60-min incubation at 37 °C. This step was repeated with 100 μL of Detection Reagent B and another 60-min incubation at 37 °C. Subsequently, 90 μL of TMB substrate was applied and incubated for 15 min at 37 °C in the dark. The reaction was stopped with 50 μL of stop solution, and absorbance was recorded at 450 nm using an Infinite 200 Pro MPlex plate reader (Tecan, Männedorf, Switzerland). SREBP-1 concentrations were calculated against standard curves ranging from 0.312 to 20 ng/mL and expressed as a percentage increase relative to controls, based on five independent triplicate experiments.

### 2.20. AMPK ELISA Kit

AMPK levels were evaluated in 3T3-L1 adipocyte lysates using the Human AMPK alpha-1,2 (Phospho) [pT172] ELISA Kit (Thermo Fisher, Milan, Italy) [64]. Lysate samples (50 μL), prepared in lysis buffer, were incubated in ELISA microplate strips on a shaker at room temperature for 60 min. Following this, a measurement reagent was added and incubated for 20 min. The enzymatic reaction was stopped by adding the stop solution, and absorbance was measured at 450 nm with an Infinite 200 Pro MPlex spectrophotometer (Tecan, Männedorf, Switzerland). Data from five independent triplicate experiments are expressed as mean ± standard deviation and reported as a percentage increase relative to the control (baseline).

### 2.21. Perilipin ELISA Kit

Perilipin 1 in 3T3-L1 adipocyte supernatants was quantified using the Human Perilipin 1 ELISA Kit (MyBiosource, San Diego, CA, USA) following the manufacturer’s protocol. After stimulation, conditioned media were collected, and cells underwent two freeze-thaw cycles and centrifugation. Samples (50 μL) were incubated with HRP-conjugate, followed by detection reagents, and absorbance was measured at 450 nm (Tecan Infinite 200 Pro MPlex). Concentrations were determined using a standard curve ranging from 0.25 to 8 ng/mL and expressed as a percentage increase compared to controls from five independent, triplicate experiments.

### 2.22. Mouse Mitochondrial Brown Fat Uncoupling Protein 1 (UCP1) ELISA Kit

Following the manufacturer’s instructions, the Cusabio ELISA kit (CUBIO Innovation Center, Houston, TX, USA) was used to measure UCP1 levels in 3T3-L1 adipocyte lysates [65]. Samples (100 μL) were incubated at 37 °C for 2 h and subsequently underwent four washes. One hundred microliters of biotinylated antibody were added and incubated for one hour at 37 °C, followed by three washes. HRP-conjugated avidin (100 μL) was incubated for 1 h at 37 °C, followed by five washes. TMB substrate (90 μL) was introduced and incubated in the dark for 15 to 30 min at 37 °C. To quantify absorbance, the reaction was stopped with 50 μL of stop solution and measured at 450 nm using a Tecan Infinite 200 Pro MPlex (Männedorf, Switzerland). UCP1 concentrations were compared to a standard curve ranging from 0 to 300 pg/mL. Data are expressed as mean ± SD, represented as a percentage of control, derived from five independent triplicate experiments.

### 2.23. Mouse PGC1 Alpha ELISA Kit

Following the manufacturer’s instructions, an ELISA kit (Antibodies, Stockholm, Sweden) was used to assess PGC1α levels in 3T3-L1 adipocyte lysates. Samples (100 μL) were incubated at 37 °C for 90 min, followed by two washes of the wells. Biotinylated detection antibody (100 μL) was added, incubated at 37 °C for 1 h, and washed three times. HRP-streptavidin conjugate (100 μL) was added and incubated at 37 °C for 1 h. After five washes, add 90 μL of TMB substrate and incubate at 37 °C for 10–20 min in the dark. To quantify the absorbance, the reaction was stopped by adding 50 μL of the stop solution and then measured at 450 nm (Infinite 200 Pro MPlex, Tecan, Männedorf, Switzerland). The concentrations of PGC1α were estimated using a standard curve ranging from 0 to 10 ng/mL. Data are expressed as mean ± SD relative to the control, derived from five independent experiments conducted in triplicate.

### 2.24. Western Blot

HepG2 and 3T3-L1 cells were lysed on ice using Complete Tablet Buffer (Roche, Basel, Switzerland) supplemented with 1 mM PMSF, 2 mM sodium orthovanadate (Na_3_VO_4_), a 1:50 dilution of phosphatase inhibitor cocktail, and a 1:200 dilution of protease inhibitor cocktail, following a standard protocol [66]. Protein extracts (35 µg) were subjected to separation via 10% SDS-PAGE, followed by transfer onto PVDF membranes (GE Healthcare Europe GmbH, Milan, Italy). Membranes were incubated overnight at 4 °C with primary antibodies diluted at 1:500 (Santa Cruz Biotechnology, Santa Cruz, CA, USA) that specifically target Resistin in HepG2 lysates and SREBP-1, AMPK, UCP1, and PGC-1α in 3T3-L1 lysates. Protein loading was assessed using an anti-β-actin antibody diluted to 1:4000 (Merck Life Science, Rome, Italy).

### 2.25. Statistical Analysis of Data

Results are presented as mean ± standard deviation, based on a minimum of five biological replicates, each conducted in triplicate. Statistical analysis was performed using one-way ANOVA followed by Bonferroni’s post hoc test or the Mann-Whitney U test, employing GraphPad Prism 5 software. Differences were considered statistically significant at *p* < 0.05, with data normalised as percentages relative to control values.

## 3. Results

### 3.1. Dose-Response Study on the Gut-Liver-Adipose Axis of Probiotics and Polycosanols Through Transwell^®^ System

**Figure 2:**
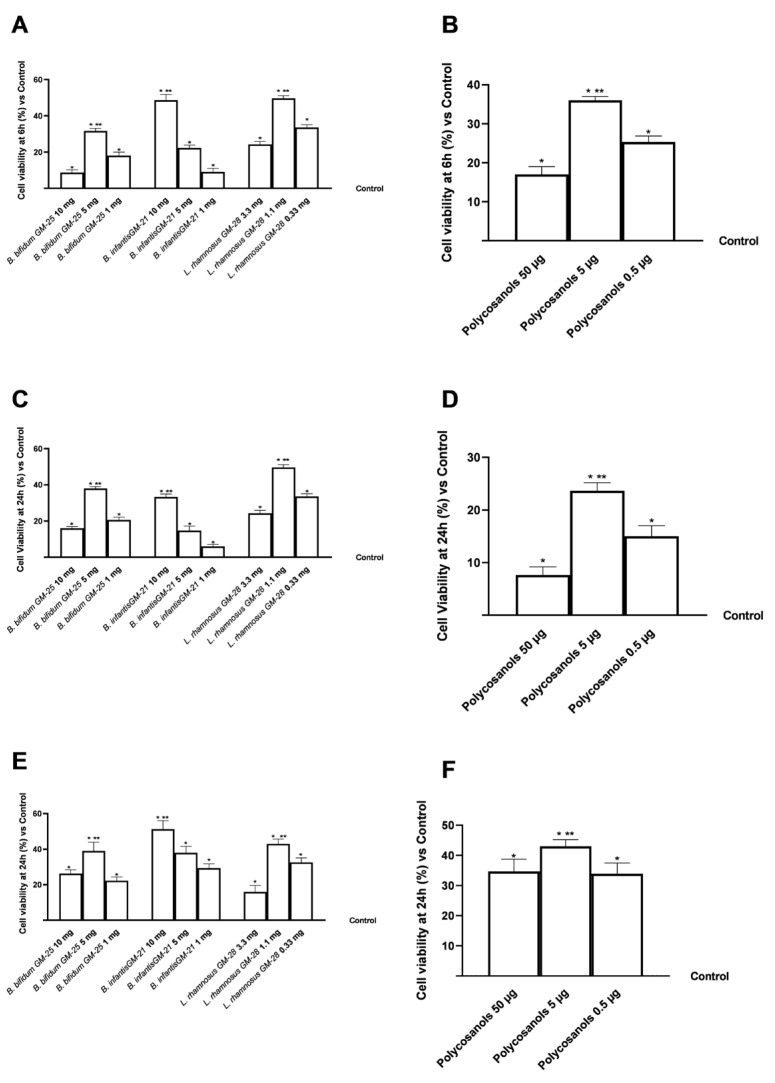
A dose-response study on cell viability on an in vitro model of gut-liver-adipose axis using respectively CaCo-2, HepG2 and 3T3-L1 pre-adipocytes cells was performed using CCK-8 assay kit. In (**A**,**B**) cell viability results of *B. bifidum* GM-25, *L. rhamnosus* GM-28, and *B. infantis* GM-21 and polycosanols on CaCo-2 cells; in (**C**,**D**) cell viability results of *B. bifidum* GM-25, *L. rhamnosus* GM-28, and *B. infantis* GM-21 and polycosanols on HepG2 cells; in (**E**,**F**) cell viability results of *B. bifidum* GM-25, *L. rhamnosus* GM-28, and *B. infantis* GM-21 and polycosanols on 3T3-L1 preadipocyte cells. Results are expressed as mean ± SD (%) of 5 independent experiments performed in triplicate vs. control (untreated sample, 0%). * *p* < 0.05 vs. control; ** *p* < 0.05 vs. other concentrations.

A preliminary screening (Figure 2) was carried out on the various cell compartments involved in the gut-liver-adipose axis on Transwell^®^ to select the best concentration in terms of maintenance and improvement of cell viability after stimulation with *B. bifidum* GM-25 (1 to 10 mg), *L. rhamnosus* GM-28 (0.33 to 3.3 mg) and *B. infantis* GM-21 (1 to 10 mg) and polycosanols (0.5 to 50 μg). As shown in Figure 1, in all 3 selected cell models all concentrations of all tested samples did not induce any cytotoxic and cell viability-reducing effect after treatment for 6 h (CaCo-2 cells, Figure 2A,B) and 24 h (HepG2 and 3T3-L1 preadipocyte cells, Figure 2C–F) with significant results than the control (*p* < 0.05) [67]. In addition, of the three concentrations tested, polycosanols 5 μg/mL maintained the most significant cell viability concerning the other concentrations (*p* < 0.05).

Overall, for the gut-liver-adipose axis model, *B. bifidum* GM-25 5 mg, *B. infantis* GM-21 10 mg and *L. rhamnosus* GM-28 1.1 mg and polycosanols 5 μg/mL were the best concentrations tested compared to other tests (*p* < 0.05), which have been used in all subsequent experiments both individually and in combination.

### 3.2. Assessment of the Effects of Probiotics and Polycosanols on an In Vitro Model of Intestinal Barrier

**Figure 3:**
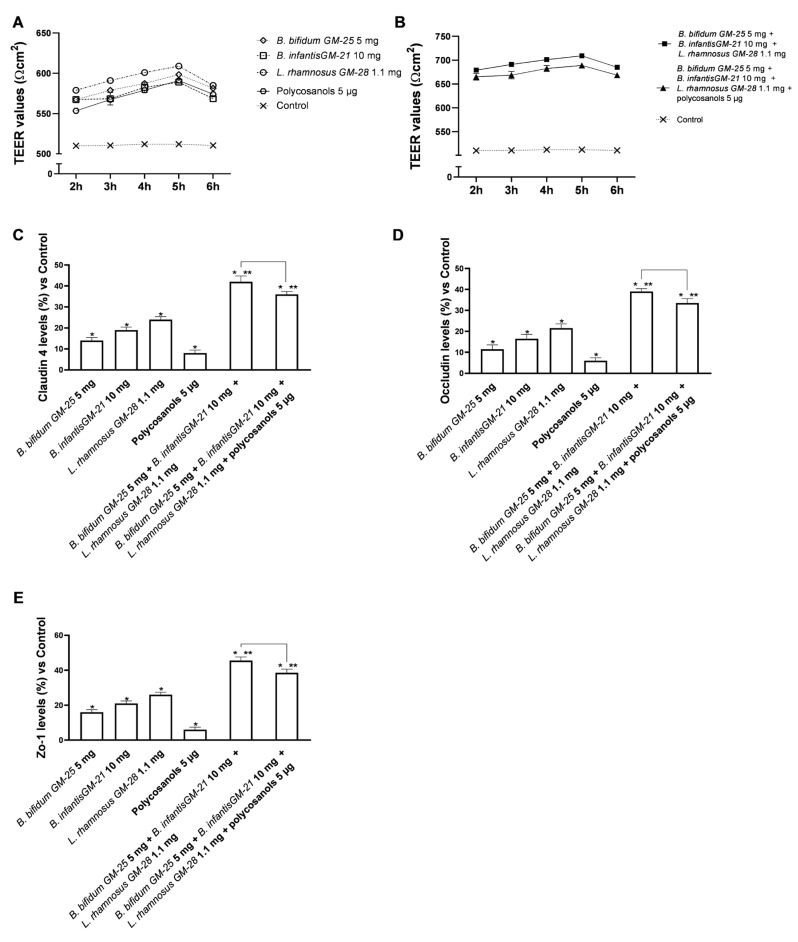
Integrity study on an in vitro model of the intestinal barrier. In (**A**,**B**) TEER analysis; in (**C**–**E**) Analysis of TJs levels by ELISA Kit. Data are expressed as mean ± SD (%) of 5 independent experiments performed in triplicate vs. control; only for (**C**–**E**), line 0 refers to the control. * *p* < 0.05 vs. control, ** *p* < 0.05 vs. to individual agents; bar *p* < 0.05 vs. the two combinations.

**Figure A1:**
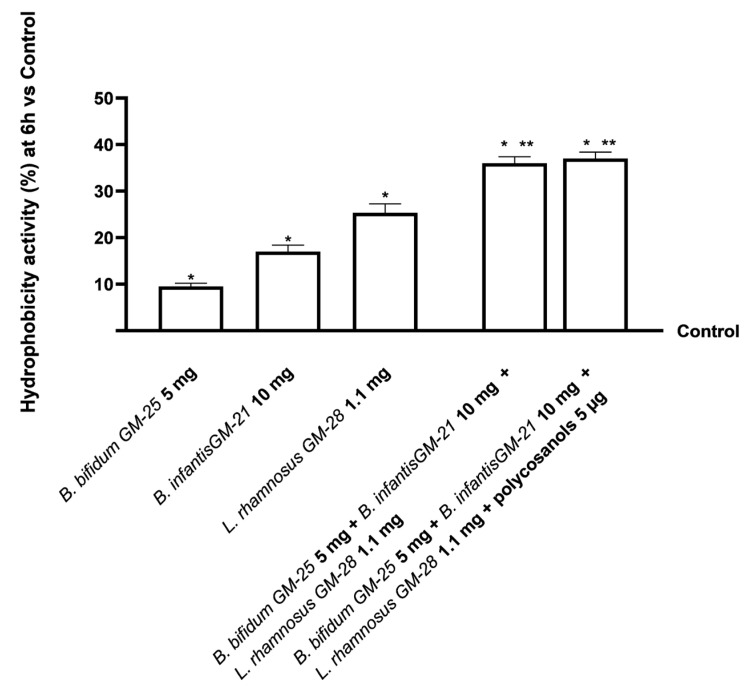
Analysis of the hydrophobicity activity of probiotics using the surface hydrophobicity assay on an in vitro model of intestinal barrier after 6 h treatment. Test results are expressed as mean ± SD (%) of 5 independent experiments performed in triplicate vs. control (0% line). * *p* < 0.05 vs. control, ** *p* < 0.05 vs. to individual agents.

To examine and study the appropriate functioning of the intestinal barrier, experiments were carried out using a 3D in vitro model that simulated the complexity of the human intestinal barrier. The effect of probiotics and polycosanols was investigated on TEER values and TJ levels for 2–6 h. As illustrated in Figure 2, the results indicate intestinal metabolism follows a physiological pattern. Investigation of the intestinal epithelium reveals that all tested agents maintained epithelial integrity (Figure 3A,B) relative to the control (*p* < 0.05) by enhancing the ion flow of paracellular exchanges across the intestinal barrier. The combination of *B. bifidum* GM-25 5 mg, *B. infantis* GM-21 10 mg, *L. rhamnosus* GM-28 1.1 mg, and polycosanols had the most substantial impact (*p* < 0.05). Adding polycosanol appears to have a statistically significant effect on the TEER identified with the probiotic mix alone, indicating their potential usage in the final formulation, particularly if they substantially impact the adipocyte or hepatic levels. The TJs examination (Figure 3C–E) supported the data provided by TEER. Specifically, Zo-1, which maintains and modifies barrier integrity, claudin, the major barrier-forming protein, and occludin, which contributes to stabilisation and optimum barrier function, were investigated. The results confirm utilising a combination created with *B. bifidum* GM-25 5 mg, *B. infantis* GM-21 10 mg, and *L. rhamnosus* GM-28 1.1 mg. Regarding Zo-1 levels, the combination of *B. bifidum* GM-25 5 mg, *B. infantis* GM-21 10 mg and *L. rhamnosus* GM-28 1.1 mg induced a 20% greater effect than the same combination with the addition of polycosanols 5 µg. Concerning claudin-4 levels, this increase is about 15%, and for occluding, about 16%. Furthermore, accurate analyses of the intestinal adhesion capacity (Figure A1 reported in Appendix A) of the probiotics under consideration, individually and in combination, have been carried out using a method validated in the literature. This permitted the verification of the surface hydrophobicity of the bacteria, associated with the ability to effectively adhere to the intestinal apical surface [59,68], after treatment of probiotics in combination also with polycosanols. The data obtained indicate that the three probiotic strains under consideration have hydrophobic cell surfaces and intestinal adhesion capacity. In addition, both combinations examined have been shown to amplify almost conspicuously the effects of this bacterial peculiarity found in the individual agents tested (*p* < 0.05).

**Figure 4:**
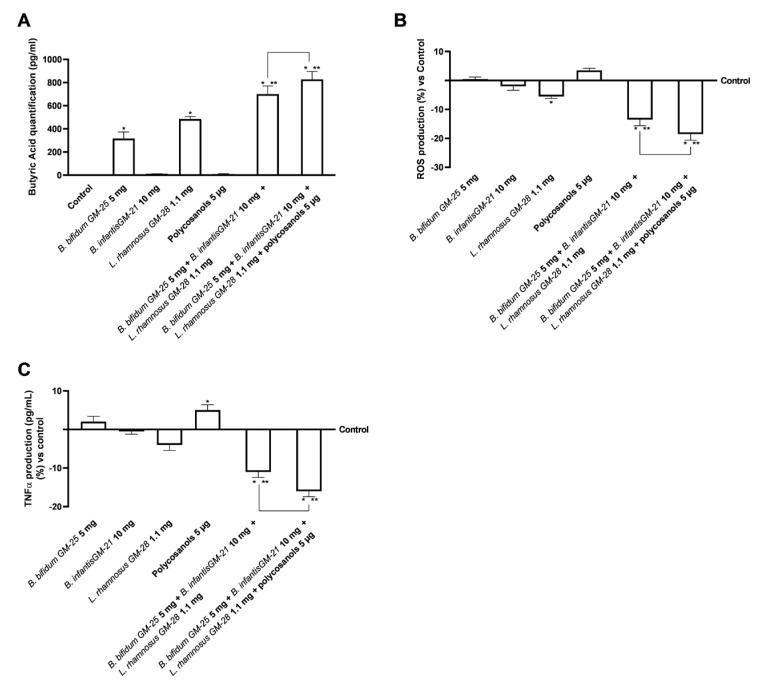
Analysis of butyrate production, oxidative stress and inflammatory effects of probiotics and polycosanols on intestinal barrier. In (**A**) Analysis of butyrate production by ELISA Kit; in (**B**) ROS production by Cytochrome C; in (**C**) TNFα production by ELISA Kit. Test results are expressed as mean ± SD (%) of 5 independent experiments performed in triplicate vs. control (0% line). * *p* < 0.05 vs. control, ** *p* < 0.05 vs. to individual agents; bar *p* < 0.05 vs. the two combinations.

Subsequently, the analysis of the amounts of butyrate produced was fundamental in the intestine following stimulations with the selected probiotics and their combination was established. Butyrate produced by gut microorganisms could play an important role in various physiological processes preventing obesity. Considering this strong connection, butyric acid production was examined after stimulation with the probiotic strains under inquiry. Specifically, as can be seen in Figure 4A, *B. infantis* GM-21 10 mg and polycosanols alone did not increase the production of butyric acid compared to the control; on the other hand, *B. bifidum* GM-25 5 mg and *L. rhamnosus* GM-28 1.1 mg improves the increase of butyric acid levels compared to control (*p* < 0.05). Further, the two combinations showed even more promising data with an increase over the control and all the single agents tested (*p* < 0.05). Specifically, in this case, the most significant effect was exerted by the combination of *B. bifidum* GM-25 5 mg, *B. infantis* GM-21 10 mg, *L. rhamnosus* GM-28 1.1 mg and polycosanols 5 µg. This combination exerted about 18% greater effect than the combination of only probiotic strains (*p* < 0.05).

Considering that oxidative stress is an essential factor contributing to obesity and metabolic syndrome [69], the production of ROS was examined on the intestinal barrier. As shown in Figure 4B, *B. infantis* GM-21 10 mg and *L. rhamnosus* GM-28 1.1 mg decreased ROS production, suggesting that the intestinal barrier was not compromised. Polycosanols increased ROS levels more than the control and the probiotics evaluated. However, this increase was insignificant; thus, they did not harm the intestinal barrier. Combining *B. bifidum* GM-25 5 mg, *B. infantis* GM-21 10 mg, *L. rhamnosus* GM-28 1.1 mg, and polycosanols enhances the effectiveness of individual agents (*p* < 0.05). This combination also decreased ROS production more effectively than the combination of *B. bifidum* GM-25 5 mg, *B. infantis* GM-21 10 mg and *L. rhamnosus* GM-28 1.1 mg (about 35%, *p* < 0.05). Finally, tumour necrosis factor α (TNFα) analysis verified the lack of intestinal inflammation after all the tested agents were stimulated (Figure 4C). Even though polycosanols alone enhanced TNFα production, this increase was just slightly superior to the control; this increase is markedly reversed when polycosanols are used in composition with probiotic strains; indeed, in this case, the most significant effect is exerted by the combination of *B. bifidum* GM-25 5 mg, *B. infantis* GM-21 10 mg, *L. rhamnosus* GM-28 1.1 mg and polycosanols 5 µg (*p* < 0.05). Specifically, this combination decreased by about 40% compared to *B. bifidum* GM-25 5 mg, *B. infantis* GM-21 10 mg and *L. rhamnosus* GM-28 1.1 mg.

### 3.3. Assessment of the Influence of Probiotics and Polycosanols on the Hepatic System Compartment

**Figure 5:**
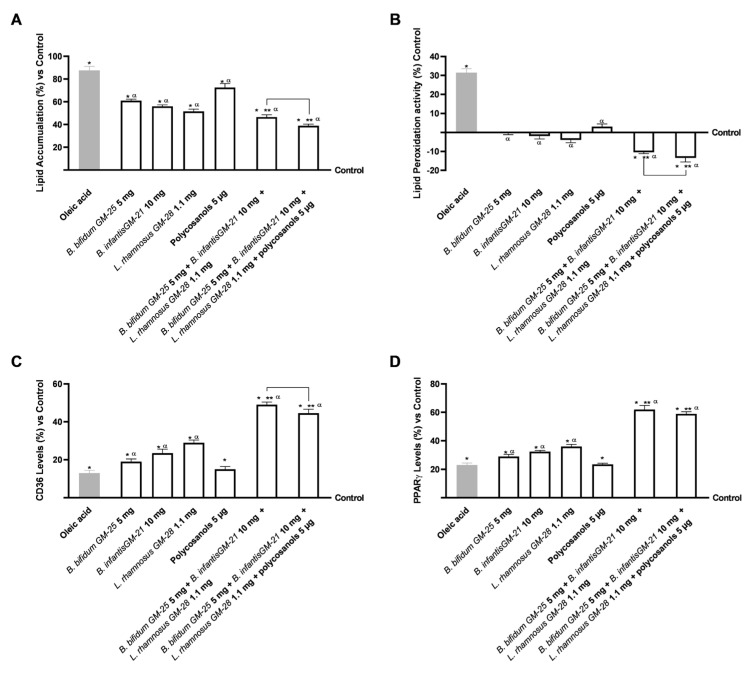
Analysis of the effects of probiotics and polycosanols on the main biomolecular mechanisms related to lipid metabolism and accumulation in the hepatic compartment after treatment with the metabolite produced after crossing the intestinal barrier. In (**A**) Lipid accumulation analysis through an adipogenesis assay kit; in (**B**) Lipid peroxidation analysis through the TBARS test; in (**C**) CD36 levels by ELISA kit; and in (**D**) PPARγ levels by ELISA kit. Test results are expressed as mean ± SD (%) of 5 independent experiments performed in triplicate vs. control (0% line). * *p* < 0.05 vs. control, α *p* < 0.05 vs. oleic acid; ** *p* < 0.05 vs. to individual agents; bar *p* < 0.05 vs. the two combinations.

**Figure A2:**
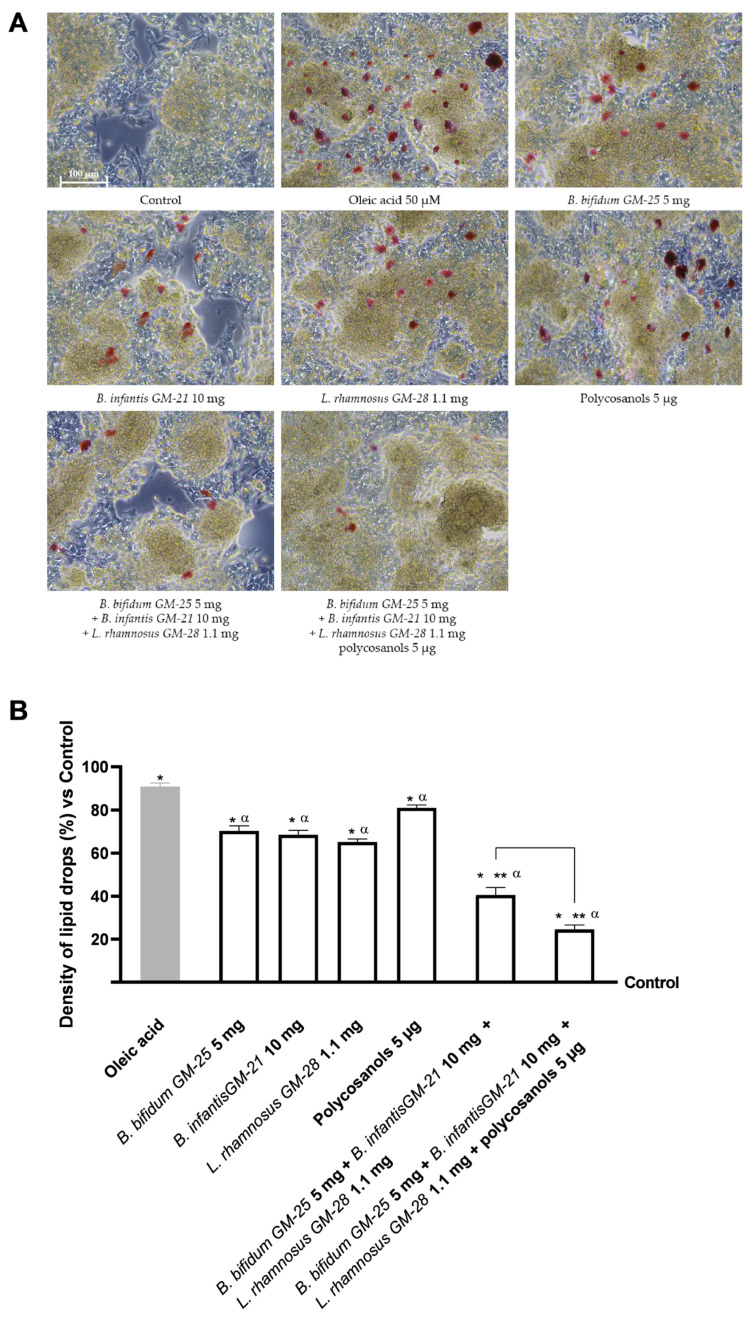
Analysis of the density of lipid drops on HepG2 pretreated with oleic acid 50 μM after stimulation for 24 h with the metabolite produced of each sample after crossing the intestinal barrier. In (**A**) the representative images for each sample were obtained at the microscope at 10× (100 μm) objective after staining with Oil Red solution; in (**B**) the % results in terms of lipid droplet density following the treatment were obtained with ImageJ software. * *p* < 0.05 vs. control, α *p* < 0.05 vs. oleic acid, ** *p* < 0.05 vs. to individual agents.

Hepatic lipid accumulation was investigated because increased body fat affects the hepatic compartment by inducing systemic metabolic dysfunction. The investigation was conducted after a 48-h pretreatment with 50 µM oleic acid to simulate in vitro metabolic dysfunction in obese subjects. After that, HepG2 cells were treated for 24 h with the basolateral medium of the intestinal Transwell^®^ system that contains the metabolite produced during the crossing intestinal barrier. Figure 5A shows that all the single compounds analysed were able to reduce the lipid accumulation caused by oleic acid pretreatment (*p* < 0.05). Even if polycosanols 5 µg alone had a minor effect, its combination with *B. bifidum G*M-25 5 mg, *B. infantis* GM-21 10 mg and *L. rhamnosus* GM-28 1.1 mg drastically increased the effects of all the compounds (*p* < 0.05). Indeed, the combination of *B. bifidum* GM-25 5 mg, *B. infantis* GM-21 10 mg, *L. rhamnosus* GM-28 1.1 mg, and polycosanols 5 µg exerted the most excellent effect with a 16% significant activity in decreasing lipid accumulation compared even to *B. bifidum* GM-25 5 mg, *B. infantis* GM-21 10 mg and *L. rhamnosus* GM-28 1.1 mg (*p* < 0.05). The results are also represented by images obtained under a microscope and the determination of lipid droplet density through ImageJ software, (Figure A2, Appendix A) after staining with Oil Red. Lipid peroxidation studies yielded comparable results, as visible in Figure 5B. All individual treatments significantly reduced oleic acid-induced inflammation (*p* < 0.05). The combination of *B. bifidum* GM-25 5 mg, *B. infantis* GM-21 10 mg, *L. rhamnosus* GM-28 1.1 mg and polycosanols 5 µg showed significant benefit compared with the single agents (*p* < 0.05). Further, the combination of *B. bifidum* GM-25 5 mg, *B. infantis* GM-21 10 mg, *L. rhamnosus* GM-28 1.1 mg and polycosanols 5 µg showed significant benefit even compared to the combination of *B. bifidum* GM-25 5 mg, *B. infantis* GM-21 10 mg and *L. rhamnosus* GM-28 1.1 mg (about 28%, *p* < 0.05). The study also investigated some signalling pathways by analysing the levels of CD36 and PPARγ after pretreatment with 50 µM oleic acid. Figure 5C shows how all the single agents, except polycosanols 5 µg, increased CD36 levels after the pretreatment. Moreover, the two combinations enhanced the single effects, obtaining the most significant results. Specifically, in this case, the best result was obtained with *B. bifidum* GM-25 5 mg, *B. infantis* GM-21 10 mg and *L. rhamnosus* GM-28 1.1 mg that induced a significant effect even compared to *B. bifidum* GM-25 5 mg, *B. infantis* GM-21 10 mg, *L. rhamnosus* GM-28 1.1 mg and polycosanols 5 µg (about 10% more, *p* < 0.05). Finally, it is known that CD36 increases transcriptional activation of the nuclear receptor PPARγ, so its levels were studied (Figure 5D). Also, in this case, almost all the single agents could increase PPARγ levels after the pretreatment (*p* < 0.05). However, polycosanols did not affect the mechanism of PPARγ compared with oleic acid. Nevertheless, its combination with probiotics strongly increases the effects (*p* < 0.05). Still, the best result was obtained with the combination of only probiotics as *B. bifidum* GM-25 5 mg, *B. infantis* GM-21 10 mg and *L. rhamnosus* GM-28 1.1 mg induce a more substantial effect even compared to *B. bifidum* GM-25 5 mg, *B. infantis* GM-21 10 mg, *L. rhamnosus* GM-28 1.1 mg and polycosanols 5 µg.

**Figure 6:**
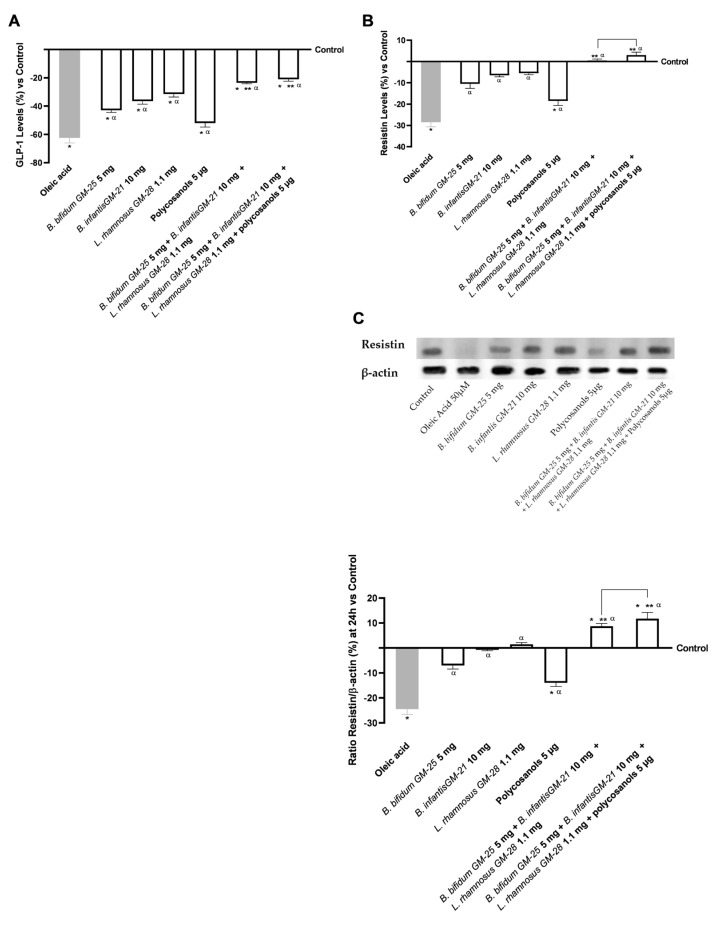
Analysis of the effects of probiotics and polycosanols on key markers related to intracellular pathways regulating insulin response and lipid accumulation on hepatic cells. In (**A**) GLP-1 levels, in (**B**) Resistin levels, all by ELISA kit and in (**C**) Resistin densitometric analysis after Western Blot, which is reported as an example image. Test results are expressed as mean ± SD (%) of 5 independent experiments performed in triplicate vs. control (0% line). * *p* < 0.05 vs. control, α *p* < 0.05 vs. oleic acid; ** *p* < 0.05 vs. to single agents; bar *p* < 0.05 vs. the two combinations.

In addition, the levels of Resistin and GLP-1 were evaluated. Figure 6A,B show that pretreatment with oleic acid reduced the levels of Resistin and GLP-1 compared with the control (*p* < 0.05). However, all agents tested, either alone or in combination, were able to mitigate this effect (*p* < 0.05). In addition, their combined use induced an even more significant effect (*p* < 0.05). Specifically, regarding GLP-1 analysis, both the combinations, with and without polycosanols 5 µg, had similar capacities in reducing the effect of oleic acid (a decrease of about 66% and 62%, respectively, *p* < 0.05). In Figure 6B, the effect of the two combinations is even more significant since they can restore the Resistin levels above the control values. In this instance, the superiority of the combination of *B. bifidum* GM-25 5 mg, *B. infantis* GM-21 10 mg, *L. rhamnosus* GM-28 1.1 mg and polycosanols 5 µg is confirmed as it induces a 5-fold increase over the other with *B. bifidum* GM-25 5 mg, *B. infantis* GM-21 10 mg and *L. rhamnosus* GM-28 1.1 mg (*p* < 0.05). By performing the Western Blot method and the densitometric analysis it was possible to confirm the data obtained through ELISA kits for Resistin. As can be seen in Figure 6C, the combination of *B. bifidum* GM-25 5 mg, *B. infantis* GM-21 10 mg, *L. rhamnosus* GM-28 1.1 mg and polycosanols 5 μg contributed to increasing the effects of individual probiotics and polycosanols (*p* < 0.05). In addition, both combinations efficiently counteracted the effect induced by 50 μM oleic acid increasing the presence of Resistin by 3.3-fold (*p* < 0.05).

### 3.4. Evaluation of the Probiotics and Polycosanols’ Influence on 3T3-L1 Adipocytes

**Figure 7:**
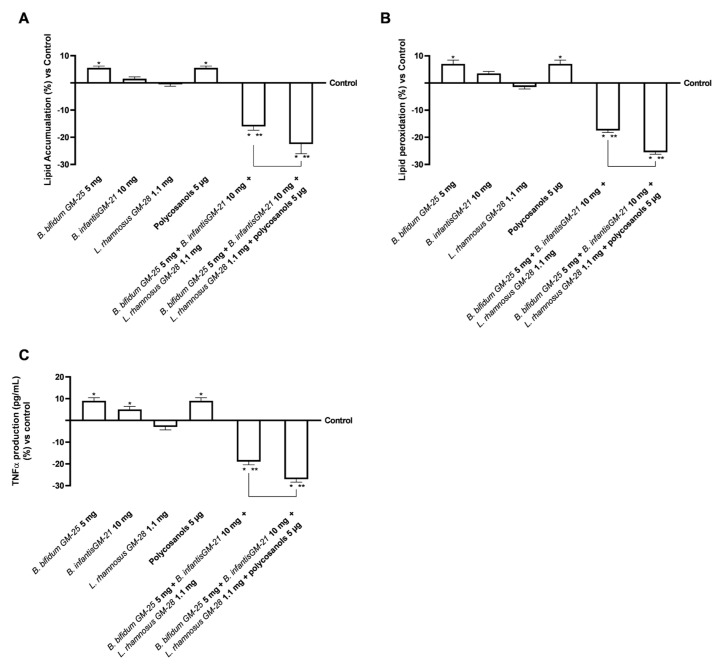
Effects of probiotics and polycosanols on 3T3-L1 adipocytes after 24 h treatment with the metabolite produced after hepatic metabolisation. In (**A**) Lipid Accumulation through an adipogenesis assay kit; in (**B**) Lipid Peroxidation through the TBARS test; and (**C**) Analysis of TNFα by ELISA kit. Test results are expressed as mean ± SD (%) of 5 independent experiments performed in triplicate vs. control (0% line). * *p* < 0.05 vs. control, ** *p* < 0.05 vs. to individual agents; bar *p* < 0.05 vs. the two combinations.

Considering the safety and efficacy of probiotics in the intestinal and hepatic environment, additional research was conducted to examine their effects on 3T3-L1 adipocytes after crossing the intestinal barrier and hepatic metabolisation. For this reason, 3T3-L1 adipocytes were treated with the basolateral medium of the intestinal Transwell^®^ system that contains the in vitro metabolite produced during the hepatic metabolisation. Then, lipid accumulation in adipose tissue was examined to determine the active role of probiotics in delaying adipogenesis. Figure 7A demonstrates that *B. bifidum* GM-25 5 mg, *B. infantis* GM-21 10 mg, and polycosanols 5 µg did not diminish lipid droplets compared to the control (*p* < 0.05). The only single agent that induced a slight decrease in lipid accumulation was *L. rhamnosus* GM-28 1.1 mg. However, even if the combination of *B. bifidum* GM-25 5 mg, *B. infantis* GM-21 10 mg and *L. rhamnosus* GM-28 1.1 mg strongly decrease the accumulation, the combination of *B. bifidum* GM-25 5 mg, *B. infantis* GM-21 10 mg, *L. rhamnosus* GM-28 1.1 mg and polycosanols 5 µg had the most significant impact (about 40% more, *p* < 0.05), indicating its effectiveness. In addition, research has shown that increased lipid peroxidation is related to obesity. As can be seen in Figure 7B, only *L. rhamnosus* GM-28 1.1 mg induced a decrease in lipid peroxidation compared to the control. However, the effect of the single agents was reverted when they were utilised in combination: both *B. bifidum* GM-25 5 mg, *B. infantis* GM-21 10 mg and *L. rhamnosus* GM-28 1.1 mg and *B. bifidum* GM-25 5 mg, *B. infantis* GM-21 10 mg, *L. rhamnosus* GM-28 1.1 mg and polycosanols 5 µg strongly decrease lipid peroxidation, with the later that exerted a 45% significant effect (*p* < 0.05). The study of TNFα activity production (Figure 7C) confirms that the new formulations do not cause inflammatory processes. The beneficial effect is enhanced through the combination formulated with *B. bifidum* GM-25 5 mg, *B. infantis* GM-21 10 mg, *L. rhamnosus* GM-28 1.1 mg and polycosanols 5 µg with a decrease of 40% compared to *B. bifidum* GM-25 5 mg, *B. infantis* GM-21 10 mg and *L. rhamnosus* GM-28 1.1 mg (*p* < 0.05).

**Figure 8:**
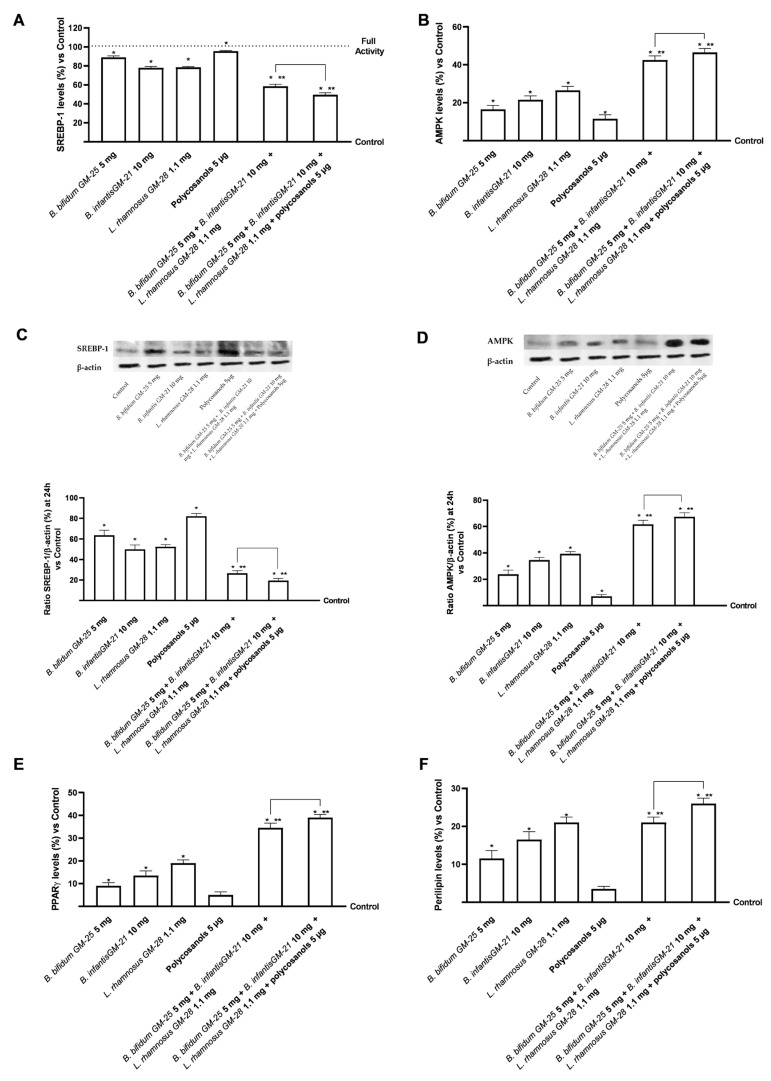
Analysis of the effects of probiotics and polycosanols on key markers involved in lipid metabolism on 3T3-L1 adipocytes after 24 h treatment with the metabolite produced after hepatic metabolisation. In (**A**) SREBP-1 levels; in (**B**) AMPK levels each other by ELISA kit; in (**C**,**D**) SREBP-1 and AMPK respectively densitometric analysis after Western Blot, which is reported as an example image; in (**E**) PPARγ levels and in (**F**) Perilipin levels, each other by ELISA kit. Test results are expressed as mean ± SD (%) of 5 independent experiments performed in triplicate vs. control (0% line). * *p* < 0.05 vs. control, ** *p* < 0.05 vs. to individual agents; bar *p* < 0.05 vs. the two combinations.

Further investigation on lipid metabolism was conducted by assessing the levels of SREBP-1, a transcription factor whose activity is dramatically increased in obese people. Figure 8A,B indicate that *B. bifidum* GM-25 5 mg, *B. infantis* GM-21 10 mg, and *L. rhamnosus* GM-28 1.1 mg significantly reduced SREBP-1 levels compared with the highest activity cut-off (*p* < 0.05). On the other hand, polycosanols 5 µg did not show significant results. However, the combination of *B. bifidum* GM-25 5 mg, *B. infantis* GM-21 10 mg, *L. rhamnosus* GM-28 1.1 mg and polycosanols 5 µg significantly reduced SREBP-1 levels compared to the single agents evaluated (*p* < 0.05) but also compared to the other combination examined (about 15% more, *p* < 0.05). In addition, Figure 8B reveals that all substances activated AMPK (*p* < 0.05), indicating their potential anti-obesity benefits. Also in this case, the combination of *B. bifidum* GM-25 5 mg, *B. infantis* GM-21 10 mg, *L. rhamnosus* GM-28 1.1 mg and polycosanols 5 µg showed the most significant effect (*p* < 0.05), with an increase of AMPK levels of 9% compared to *B. bifidum* GM-25 5 mg, *B. infantis* GM-21 10 mg and *L. rhamnosus* GM-28 1.1 mg (*p* < 0.05). For both biological markers described above, studies were conducted in terms of densitometric analysis in cell lysats following the Western Blot (Figure 8C,D). The data shown confirm that the combination of *B. bifidum* GM-25 5 mg, *B. infantis* GM-21 10 mg, *L. rhamnosus* GM-28 1.1 mg and policosanols 5 μg showed the most significant effect (*p* < 0.05), with a reduction in the presence of SREBP-1 of 65% compared to B. bifidum GM-25 5 mg, *B. infantis* GM-252521 10 mg and *L. rhamnosus* GM-28 1.1 mg (*p* < 0.05). Regarding AMPK, the combination of *B. bifidum* GM-25 5 mg, *B. infantis* GM-21 10 mg, *L. rhamnosus* GM-28 1.1 mg and policosanols 5 μg showed the most significant effect (*p* < 0.05), with an increase in the presence of the biological marker of 50% compared to *B. bifidum* GM-25 5 mg, *B. infantis* GM-252521 10 mg and *L. rhamnosus* GM-28 1.1 mg (*p* < 0.05).

As mentioned above, obesity is characterised by persistent low-grade inflammation. In this context, PPARγ regulates pro-inflammatory genes in the liver and adipose tissue, resulting in anti-inflammatory effects. Polycosanols 5 µg alone did not substantially affect PPARγ activation compared with control. However (Figure 8E), the combination of *B. bifidum* GM-25 5 mg, *B. infantis* GM-21 10 mg, *L. rhamnosus* GM-28 1.1 mg and polycosanols 5 µg had the highest efficacy compared with all the single agents and, particularly, also with the same combination without polycosanols (about 13%, *p* < 0.05). This data was further confirmed by analysis of perilipin levels (Figure 8F), modulated by activation in 3T3-L1 adipocytes. Indeed, although all substances alone can modulate its activation compared with control (*p* < 0.05), the least effect is observed following treatment with Polycosanols 5 µg. As expected, the formulation of *B. bifidum* GM-25 5 mg, *B. infantis* GM-21 10 mg, *L. rhamnosus* GM-28 1.1 mg and polycosanols 5 µg can induce a significant increase in the individual agents (*p* < 0.05); in addition, the formulation of *B. bifidum* GM-25 5 mg, *B. infantis* GM-21 10 mg, *L. rhamnosus* GM-28 1.1 mg and polycosanols 5 µg also induces a significant increase in perilipin levels compared to the other combination under investigation (about 24%, *p* < 0.05).

**Figure 9:**
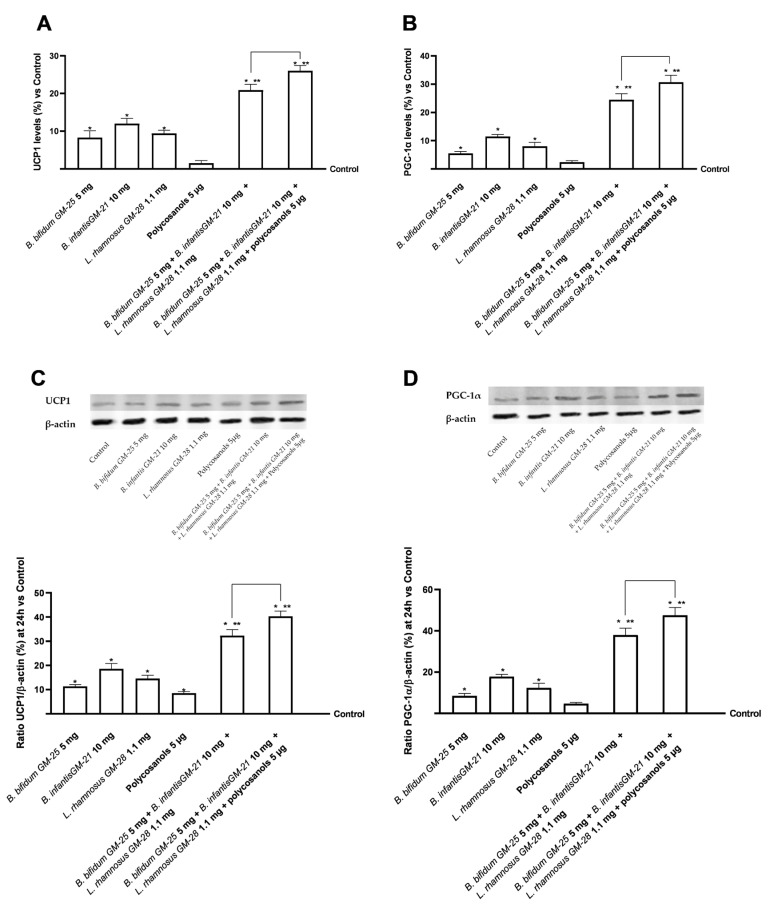
Analysis of the effects of probiotics and polycosanols on key markers browning signalling in 3T3-L1 white adipocytes after 24 h treatment with the metabolite produced after hepatic metabolisation. In (**A**) UCP1 levels; in (**B**) PGC-1α levels all by ELISA kit; in (**C**,**D**) UCP1 and PGC-1α respectively densitometric analysis after Western Blot, which is reported as an example image. Test results are expressed as mean ± SD (%) of 5 independent experiments performed in triplicate vs. control (0% line). * *p* < 0.05 vs. control, ** *p* < 0.05 vs. to individual agents; bar *p* < 0.05 vs. the two combinations.

In the last part of the 3T3-L1 adipocyte line research phase, the effects of converting WAT to beige adipocytes with BAT-like characteristics were examined. The study focused on key proteins of the BAT phenotype and thermogenesis, such as UCP1 and PGC-1α, which allow the targeting of adipose fat consumption to produce energy. As shown in Figure 9A, after treatment for 24 h with the test samples and their combinations, UCP1 activity was increased compared with control in adipocyte cells with statistically significant values only after treatment with probiotics (*p* < 0.05). Greater data were obtained after treatment with liver metabolised associated with *B. infantis* GM-21 10 mg. The combination of *B. bifidum* GM-25 5 mg, *B. infantis* GM-21 10 mg, and *L. rhamnosus* GM-28 1.1 mg was shown to improve the effects of individual probiotics significantly (*p* < 0.05). Adding polycosanols 5 µg amplified the effects of the combination of the three probiotics examined in detail by 25% (*p* < 0.05). Comparable results were obtained regarding the expression of PGC-1α levels following treatment with the test samples. Again, stimulation with hepatic metabolised individual probiotics improved the levels of the protein marker compared with control 3T3-L1 adipocyte cells (*p* < 0.05), with better data from *B. infantis* GM-21 10 mg. Both combinations examined improved the effects of individual probiotics and polycosanols 5 µg (*p* < 0.05) with more significant data from the combination of *B. bifidum* GM-25 5 mg, *B. infantis* GM-21 10 mg, *L. rhamnosus* GM-28 1.1 mg and polycosanols 5 µg (about 27%, *p* < 0.05).

After the Western Blot, further analyses were performed using densitometric analysis in cell lysates for both biological markers mentioned above (Figure 9C,D). Compared to *B. bifidum* GM-25 5 mg, *B. infantis* GM-252521 10 mg, and *L. rhamnosus* GM-28 1.1 mg, the combination of *B. bifidum* GM-25 5 mg, *B. infantis* GM-21 10 mg, *L. rhamnosus* GM-28 1.1 mg and polycosanols 5 μg increased the presence of UCP1 by 68% according to the data (*p* < 0.05). Similar results were also seen for PGC-1α, where the most significant impact (*p* < 0.05) was demonstrated by the combination of *B. bifidum* GM-25 5 mg, *B. infantis* GM-21 10 mg, *L. rhamnosus* GM-28 1.1 mg, and polycosanols 5 μg. Similar effects were also found for PGC-1a where the combination of *B. bifidum* GM-25 5 mg, *B. infantis* GM-21 10 mg, *L. rhamnosus* GM-28 1.1 mg and polycosanols 5 μg showed the most significant effect (*p* < 0.05), with a 71% increase in the presence of the biological marker compared to *B. bifidum* GM-25 5 mg, *B. infantis* GM-252521 10 mg and *L. rhamnosus* GM-28 1.1 mg (*p* < 0.05).

## 4. Discussion

One of the biggest health issues of the twenty-first century, obesity, is categorised as a worldwide epidemic and has become a serious public health concern [1]. It is influenced by environmental, genetic, neuronal, endocrine, and behavioural factors. Environmental factors, genetic predisposition, food, and inactivity contribute to obesity and other chronic non-communicable diseases [70,71]. The gut-liver-adipose tissue axis is closely linked to lipid accumulation, which can reduce the liver’s ability to extract substances from the gut and alter microsome function [72]. This connection is crucial for the intestinal microbiota [73]. Obese patients often have dysbiosis and decreased microbial diversity in their gut microbiota, encouraging more energy absorption from food and making it easier for adipose tissue to store energy [74].

A reduction in fasting-induced adipose factor is another effect of this altered microbiota. Due to this suppression, adipose tissue stores more triglycerides and releases fewer hormones that encourage food intake, such as GLP-1. There are strong signs that the gut microbiota is crucial for preserving the balance of energy metabolism despite the paucity of human studies in this area [74,75]. Furthermore, the alteration of gut microbiota composition is directly associated with enhanced release of the hormone GLP-1. Probiotics have been demonstrated to increase butyrate and other SCFA levels, which causes intestinal L-cells to release more GLP-1. These data indicate a probiotic–gut microbiota–butyrate–GLP-1 axis that improves metabolic function and protects against high-fat diet-induced obesity and diabetes without using GLP-1 agonists and their side effects [76]. Further study in adipose tissue biology, notably on the molecular mechanisms regulating adipocyte differentiation and adipogenesis, supports these findings [77]. In vitro cell models and molecular biology methods can reveal the complicated processes of adipocyte commitment and differentiation, revealing more about adipogenesis and obesity-related adipocyte dysfunction [78]. Numerous studies attempt to improve the various human and animal cell culture models available for studying the process of adipogenic differentiation in vitro concerning obesity and associated co-morbidity [79]. The primary characteristics, novel techniques, and potential uses of cell models to research adipogenesis have been extensively changed over the past five years. Additionally, three-dimensional culture models and co-culture methods provide novel platforms for examining the interactions between adipocytes and nearby cells in adipose tissue [80]. Numerous experimental models have been developed to investigate the molecular and biological factors behind obesity. For various reasons, studying obesity-related pathways in vitro requires using cellular models, including 3T3-L1 and HepG2 cells [81]. 3T3-L1 cells, classified as adipogenic fibroblasts, can be used to research adipocyte differentiation and lipid metabolism. These cells offer a great method for investigating external stimuli, such as nutrition and hormones, that impact fat accumulation [82]. Instead, HepG2 cells are used as a model of human hepatocytes, making it possible to research liver metabolism, a vital organ for maintaining energy balance. These models may be used to investigate the mechanisms of insulin resistance, hepatic steatosis, and changes in glucose and lipid metabolism associated with obesity [83]. As a result, the study focused on using cellular models to improve and incentivise scientific research in this area. Therefore, the modulation mechanisms of lipid accumulation exerted by the agents under investigation were analysed using cellular models of the intestine, liver and adipose tissue.

First, the intestinal barrier-level effects of the three probiotics combined to preserve the stability and proper functioning of the replicated intestinal barrier in vitro by regulating intestinal flow exchange were described. Compared to single agents, the combination of *B. bifidum* GM-25 5 mg, *B. infantis* GM-21 10 mg and *L. rhamnosus* GM-28 1.1 mg preserved intestinal integrity and function in terms of the examined TJs, such as claudin 4, which maintains the structure, occludin, which helps with stabilisation, and Zo-1, which mediates adhesion. Adding polycosanols did not increase the effect of the three probiotics combined but still preserved the superior efficacy of the probiotics and polycosanols tested individually. Consistent results were obtained in terms of hydrophobicity and adhesion ability of probiotics. The selected assay was useful for studying interactions with the intestinal epithelium, allowing their possible maintenance at that site following oral intake to be studied in vitro. As also demonstrated in the literature [59,68], it was possible to characterise the hydrophobic surface of the bacteria associated with the ability to efficiently adhere to the apical surface by *B. bifidum* GM-25 5 mg, *B. infantis* GM-21 10 mg and *L. rhamnosus* GM-28 1.1 mg, with better data from *L. rhamnosus* GM-28 1.1 mg (Figure A1 reported in Appendix A). The combination of *B. bifidum* GM-25 5 mg, *B. infantis* GM-21 10 mg and *L. rhamnosus* GM-28 1.1 mg amplified this examined parameter suggesting an enhanced ability to colonise and protect intestinal barrier integrity, which was maintained even after the addition of polycosanols 5 μg with no statistically significant difference. At the same time, the combination of *B. bifidum GM-25* 5 mg, *B. infantis GM-21* 10 mg, *L. rhamnosus GM-28* 1.1 mg and polycosanols 5 µg also produced favourable results in terms of butyrate production, and reduction of inflammatory profile trigger (*p* < 0.05). Butyrate, produced by the intestinal bacteria, may regulate gut physiological activities [84]. Butyrate controls transepithelial fluid transport by lowering mucosal inflammation, visceral sensitivity, and intestinal motility. Recent research suggests that butyrate, whether endogenous or exogenous, can prevent obesity and metabolic diseases [85]. According to randomized clinical research, oral butyrate supplementation can alleviate pediatric obesity [86].

Considering how increasing body fat impacts the hepatic compartment by encouraging systemic metabolic dysfunction, assessing lipid accumulation at the hepatic level was equally interesting. Specifically, after 48 h of pretreatment with 50 µM oleic acid to simulate metabolic inability in obese people, all probiotics under research showed decreased lipid accumulation levels. The use of the combination with polycosanols 5 µg further accentuates this improvement. Analysis of lipid peroxidation also showed similar results. All individual agents were able to significantly decrease the inflammatory mechanisms induced by oleic acid (*p* < 0.05). In addition, the beneficial effect induced by the combination formulated with the combination of *B. bifidum* GM-25 5 mg, *B. infantis* GM-21 10 mg, *L. rhamnosus* GM-28 1.1 mg and polycosanols 5 µg over the others has been further confirmed (*p* < 0.05). In addition, connected with the state of lipid peroxidation, it has been defined that the expression of CD36 in liver tissue may contribute to the development of inflammation [87,88]. In other studies, CD36 facilitated the uptake of long-chain fatty acids and oxidized LDL and there is evidence that CD36 also plays a role in lipolysis [89,90,91,92]. The probiotics examined alone could modulate CD36 levels compared with oleic acid (*p* < 0.05). In addition, their use combined with polycosanols 5 µg induced a cooperative effect by modulating CD36 levels (*p* < 0.05). They also modulate the anti-inflammatory effect of PPARγ by increasing it [93,94], a mechanism directly dependent on and consistent with those obtained on CD36, which promotes transcriptional activation of the nuclear receptor PPARγ. Finally, resistin and GLP-1 levels were examined for their roles in obesity and insulin resistance. They showed their importance in cholesterol metabolism [88,95] and body weight reduction by promoting satiety and delaying gastric emptying. Consistent with what has been defined by the previous results, the combination of *B. bifidum* GM-25 5 mg, *B. infantis* GM-21 10 mg, and *L. rhamnosus* GM-28 1.1 mg with and without polycosanols 5 µg reduced the pro-obesogenic effect promoted by oleic acid pretreatment more than the singles and considerably annihilated about resistin activation levels in hepatocytes (*p* < 0.05). This was also confirmed by densitometric analysis; indeed, the presence of Resistin in HepG2 lysates was greatly increased after treatment with both combinations compared to individual agents (*p* < 0.05).

Finally, in relation to the close gut-liver-adipose tissue connection, the effects of examination samples at the level of differentiated adipocytes after hepatic metabolisation were examined. The combination of *B. bifidum* GM-25 5 mg, *B. infantis* GM-21 10 mg, *L. rhamnosus* GM-28 1.1 mg and polycosanols 5 µg produced favourable results in terms of low decreased fat buildup and decreased inflammatory profile trigger (*p* < 0.05). These findings attest to the ability of combinations to lessen adipocyte-level lipid buildup. The close connection between the state of lipid peroxidation and the inflammatory profile has depended mainly on measuring oxidative stress levels and activating the marker [96]. Numerous studies show that reducing obesity leads to lower lipid peroxidation and downregulation of genes in the tumour necrosis factor α/nuclear factor kappa-light-chain-enhancer of activated B cells (TNFα/NF-κB) signalling pathway [97,98]. Kuennen’s (2012) [98] study devotes a significant portion to exploring inflammation and how it is affected by excessive lipid accumulation and obesogenic profile. Chronic low-grade inflammation is common in obese people because adipose tissue generates pro-inflammatory cytokines and stores energy. These mediators, which include C-reactive protein, interleukin-6 (IL-6), and TNF-α, raise the risk of developing chronic diseases like type 2 diabetes and cardiovascular disease [98]. Studies using 3T3-L1 adipocytes treated with palmitate have shown that nutraceutical interventions can reduce the expression of TNF-α and IL-6 induced by palmitate. The inhibition of NF-κB and Jun N-terminal kinases (JNK) signalling pathways, which are normally triggered in response to saturated fatty acids, mediates this anti-inflammatory action. As a result, this approach attenuates inflammation in adipose tissues, thereby reducing the risk of insulin resistance and other obesity-related metabolic complications [99,100,101]. Examining the primary markers associated with obesity and adiposity, including SREBP-1, AMPK, PPARγ, and perilipin, has provided promising evidence for these findings. SREBP-1 is a transcription factor mainly expressed in muscle, adipose, and liver tissue that has markedly increased activity in fat people. Indeed, it is a key driver of lipid accumulation in hepatocytes and adipocytes, and its overexpression is associated with obesity, hepatic steatosis, and metabolic inflammation [102]. Here, it can activate the genes glucokinase, exokinase, and those that code for synthesising fatty acids. At the same time, lipid metabolism is also influenced by AMPK, an essential regulator of energy metabolism. It regulates lipogenesis and lipolysis in adipose tissue, and its activation in adipocytes leads to a decrease in the absorption of fatty acids, a reduction in triglyceride synthesis and increased fatty acid oxidation and inhibition of lipogenic gene expression [103]. AMPK activation counteracts obesity-related metabolic dysfunction by inhibiting SREBP-1 in 3T3-L1 adipocytes [104,105]. The results show how the formulation of *B. bifidum* GM-25 5 mg, *B. infantis* GM-21 10 mg, and *L. rhamnosus* GM-28 1.1 mg with and without polycosanols improves the metabolic and lipid profile at the level of the adiposity compartment. In addition to the individual agents (*p* < 0.05), the examined combinations have strengthened the obtained data regarding the less inflammatory state present. In addition, in terms of densitometric analysis after Western blot the presence of SREB-1 was widely reduced in the 3T3-L1 lysates after treatment with both combinations compared to the individual agents (*p* < 0.05); while at the same time, the presence of AMPK was increased (*p* < 0.05).

Low-grade chronic inflammation characterises obesity. In this context, PPARγ is a nuclear receptor that orchestrates adipocyte differentiation, lipid storage, and glucose metabolism [106,107]. It plays a fundamental role as it modulates the pro-inflammatory genes in the liver and adipose tissue, thus performing an anti-inflammatory action. This has been most activated in adiposity following stimulation with the combination of *B. bifidum* GM-25 5 mg, *B. infantis* GM-21 10 mg, and *L. rhamnosus* GM-28 1.1 mg with and without polycosanols 5 µg. Furthermore, perilipin levels are influenced by PPARγ activation in 3T3-L1 adipocytes, supporting the results. Pyrilipine has essential roles in the control of basal and hormonal lipolysis. Treatment with *B. bifidum* GM-25 5 mg, *B. infantis* GM-21 10 mg, *L. rhamnosus* GM-28 1.1 mg and polycosanols 5 µg significantly increased perilipin levels compared to the other test samples (*p* < 0.05).

Ultimately, the study also allowed us to examine the effect of individual agents and combinations, not only on adipogenesis but also on the 3T3-L1 adipocyte browning process. This process is a key step in reducing lipid accumulation, promoting the utilisation of excess fat for thermogenesis which has also been observed in natural extracts and probiotics studies [17,108,109,110,111]. Studies have shown that WAT-type adipocytes switch to a beige phenotype when they acquire BAT-like characteristics [17,110,112]. This type of phenotypic transition was evidenced in studies in which AMPK was more activated at the level of 3T3-L1 adipocytes [106] AMPK acts as an energy sensor by directing intracellular fat consumption for energy purposes, as verified in our study by the probiotic and polycosanol combinations examined [105,113]. In addition to this, to better elucidate the mechanism underlying the anti-obesity effects of *B. bifidum* GM-25 5 mg, *B. infantis* GM-21 10 mg, and *L. rhamnosus* GM-28 1.1 mg and polycosanols 5 µg, BAT marker proteins such as PGC-1α and UCP1 involved in adaptive thermogenesis and the transition of WAT to BAT were also examined. For both markers, both combinations amplified the effects of individual probiotics and polycosanols in terms of increased UCP-1 activity and PGC-1α levels. The best effects were observed after adding polycosanols in the combination of *B. bifidum* GM-25 5 mg, *B. infantis* GM-21 10 mg, and *L. rhamnosus* GM-28 1.1 mg and polycosanols 5 µg. Consistent results for both UCP1 and PGC-1α were obtained following the densitometric analysis on the cell lysates of 3T3-L1 adipocytes confirming when obtained by ELISA kit. These data suggest that due to intestinal and hepatic metabolism, probiotic metabolites and polycosanols have contributed to mitochondrial biogenesis and energy metabolism.

Although our study presents an in vitro physiologically relevant model of the gut-liver-adipose axis to study the anti-obesity potential of a probiotic-policosanol combination, it is worth recognizing some limitations. First, the model, although multi-compartment, does not fully capture the systemic complexity of human physiology. The absence of interactions with the immune, endocrine and nervous systems may influence the translational relevance of the results. Secondly, in vitro models (CaCo-2, HepG2 and 3T3-L1) have been used, which, although well validated for permeability, metabolic and adipogenic studies, may not fully reflect the primary cellular behaviour or inter-individual variability observed in vivo. Third, the study was limited to short-term exposure and acute responses; therefore, long-term effects, potential coping mechanisms or toxicity were not assessed. Also, while we have observed significant changes in the main markers of fat metabolism, inflammation and adipocyte browning, no mechanistic studies (for example transcriptomic profiling) were performed and should be addressed in future work. In the future, in vivo studies and clinical trials will be essential to validate the efficacy and safety of this formulation and its relevance in preventing or treating obesity-related disorders.

## 5. Conclusions

By establishing an in vitro model of liver-adipose tissue, our research discovered that the impact of probiotics on lipid metabolism and accumulation varies by strain and that only a few species belonging to the genera *Lactobacillus* and *Bifidobacterium* may demonstrate efficacy. More research is needed to determine probiotics’ ideal dosage, timing, and long-term efficacy in preventing overweight and obesity. Based on the results gathered from this study, it is evident how the combination of polycosanols and probiotics might enhance the recognised mechanisms that combat obesity and reduce the mechanisms that instead contribute to its advancement. In addition, the combination helps to direct towards higher fat consumption at the mitochondrial level by contributing to thermogenesis and the transition to an adipose beige phenotype.

## Figures and Tables

**Table 1:** Concentration was utilised, and CFU-mg was converted for each probiotic strain.

Probiotic Strain	Accession Number	Concentration in CFU	Concentration in mg
*Bifidobacterium bifidum GM-25*	DSM 34624	1 × 10^8^ CFU–1 × 10^9^ CFU	1 mg–10 mg
*Bifidobacterium infantis GM-21*	DSM 34621	1 × 10^8^ CFU–1 × 10^9^ CFU	1 mg–10 mg
*Lacticaseibacillus rhamnosus GM-28*	DSM 34619	1 × 10^8^ CFU–1 × 10^9^ CFU	0.33 g–3.3 mg

## Data Availability

The original contributions presented in the study are included in the article, further inquiries can be directed to the corresponding author.

## Abbreviations

The following abbreviations are used in this manuscript:

AMPK	AMP-activated protein kinase
aP2	adipose-specific fatty acid-binding protein
ATCC	American Type Culture Collection
BAT	Brown Adipose Tissue
BMI	body mass index
C/EBP-α	CCAAT/enhancer-binding protein-α
C/EBPβ	CCAAT/enhancer-binding protein-β
C/EBPδ	CCAAT/enhancer-binding protein-δ
CCK8	Cell Counting Kit 8
CD36	cluster of differentiation 36
CFU	colony forming unit
CPT-1	carnitine palmitoyltransferase-1
DMEM	Dulbecco’s Modified Eagle’s Medium
DMEM-F12	Dulbecco’s Modified Eagle’s Medium/Nutrient F-12 Ham
ELISA	Enzyme-Linked Immunosorbent Assay
EVOM3	Epithelial Volt/Ohm Meter
FAS	fatty acid synthase
FBS	foetal bovine serum
FFA	free fatty acids
GLP-1	glucagon-like peptide 1
IL-6	interleukin-6
JNK	Jun N-terminal kinases
LC3β	microtubule- associated proteins 1A/1B light chain 3B
NF-kB	nuclear factor kappa-light-chain-enhancer of activated B cells
PBS	Phosphate Buffer Saline
PCSK9	Proprotein Convertase Subtilisin/Kexin type 9
PGC-1α	Peroxisome Proliferator Activated Receptor 1 Alpha
PPAR-γ	Peroxisome activated receptor-γ
ROS	reactive oxygen species
SCFA	short-chain fatty acid
SREBP-1	sterol regulatory element-binding protein-1
SREBP-1c	sterol regulatory element-binding protein-1c
TEER	Trans-epithelial electric resistance
TJ	tight junctions
TNFα	tumor necrosis factor α
UCP1	uncoupling protein 1
WAT	White Adipose Tissue
YB	*Bifidobacterium longum* subsp. *infantis* YB0411
